# Clinical laser treatment of toenail onychomycoses

**DOI:** 10.1007/s10103-017-2198-6

**Published:** 2017-04-04

**Authors:** Antonio Zalacain, Alejandra Merlos, Elena Planell, Erica G. Cantadori, Teresa Vinuesa, Miguel Viñas

**Affiliations:** 1 Department of Pathology and Experimental Therapeutics, Campus de Ciències de la Salut de Bellvitge, University of Barcelona, Barcelona, Spain; 2 Department of Podiatry, Campus de Ciències de la Salut de Bellvitge, University of Barcelona, Barcelona, Spain

**Keywords:** Onychomycosis, Toenail fungal infection, Laser 1064 nm

## Abstract

Onychomycoses are fungal infections of the fingernails or toenails having a prevalence of 3% among adults and accounts for 50% of nail infections. It is caused by dermatophytes, non-dermatophyte filamentous fungi, and yeasts. Compressions and microtraumas significantly contribute to onychomycosis. Laser and photodynamic therapies are being proposed to treat onychomycosis. Laser light (1064 nm) was used to treat onychomycosis in 156 affected toenails. Patients were clinically followed up for 9 months after treatment. Microbiological detection of fungal presence in lesions was accomplished. A total of 116 samples allowed the isolation of at least a fungus. Most of nails were affected in more than two thirds surface (some of them in the full surface). In 85% of cases, after 18 months of the onset of treatment, culture turned negative. After 3 months months, only five patients were completely symptom-free with negative culture. In 25 patients, only after 6 months, the absence of symptoms was achieved and the cultures negativized; in 29 patients, 9 months were required. No noticeable adverse effects were reported. This study reinforces previous works suggesting the applicability of laser therapies to treat toenail onychomycosis.

## Introduction

Fungal infection of the toenails (onychomycosis) has a prevalence of 3% accounting for 50% of nail infections [1–3]. The causative microorganisms are dermatophytes, non-dermatophyte filamentous molds, or yeasts. Compressions and microtraumas are the main triggers of onychomycosis of the toes which are frequent in diabetics and immunocompromised individuals, as well as in those suffering peripheral arterial diseases [4]. Athletes and workers wearing protective metal toe-capped shoes tend to develop toenail injuries that facilitate infection [5, 6]. It is often treated with either local or systemic antifungals, but recurrence and treatment failure are common; thus, therapeutic alternatives are needed [7]. Antifungal agents, such as terbinafine, itraconazole, and fluconazole, combined with the avulsion or debridement are the gold standard of treatment (healing rates between 40 and 80%) [8, 9]. However, the high toxicity of antifungals as well as interaction of azols with other drugs are a matter of concern such that their use in patients who are immunocompromised, including newly transplanted patients, those undergoing chemotherapy, and medicated patients, is not recommended despite these individuals are at serious risk of fungal infection [10]. Focuses of current research into the treatment of onychomycosis look for shorter duration of therapy in the form of a pulse regimen and the use of topical antifungal lacquers. Index of topical therapy is low, particularly when nail involvement is greater than 50%.

The neodymium-doped yttrium aluminum garnet (Nd-YAG) laser at a wavelength of 1064 nm, emitting through a pulsed-beam system and with fiber delivery, has been shown to be useful in dermatology, dentistry, and some other medical specialties. It is used to treat onychomycoses and warts, and in some other treatments. Temperature increases moderately and almost no inflammatory responses have been reported, either immediately or after 30 days following the use of such a laser.

During the last few years, several novel therapeutic approaches have been investigated, including photodynamic therapy, iontophoresis, and ultrasound; however, no clear progress in any of them has been reported [11]. An alternative therapeutic approach for treatment of onychomycosis, yielding promising results, is 1064-nm laser. Among its main advantages is that it can be used in almost all patients, has no associated side effects, and avoids the need for systemic antifungals.

The use of laser to treat onychomycosis in a relevant number of patients and with a follow-up of 12 months was first reported in 2010 [12]. Significant success rates were achieved with 870- and 930-nm lasers. In 2010, Landsman et al. [13] demonstrate that laser treatment (870–930 nm) is reliable and safe, reflecting the fact that the mechanism of action for fungal inactivation is purely photobiological, and that such a treatment does not depend on ablation, high heat, chemical potentiators, or any wavelength in the ultraviolet range. Two years later [14], it was shown that such wavelengths gave a complete mycological cure at 270 days on 38% of treated individuals. For these reasons, in our study, patients with onychomycosis were treated with 1064-nm laser; the treatment success rate was 83.7%. It should be stated that our population had a higher degree of severity than that of Landsman et al. Since 2011, several studies focusing on the treatment of onychomycosis with 1064-nm laser have been published [15, 16], including those in which only the fingernails were treated [17, 18]. In some studies, different wavelengths were combined and the number of sessions reduced. For example, in one study, both 1064- and 532-nm laser were used in alternative sweeps, with only two sessions performed [19, 20]. In general, the results of all studies published show that the efficacy of 1064-nm laser is higher than that of other forms of treatment. However, general recommendations are not possible, given the great diversity of laser treatment protocols and the lack of reliable data concerning laser-beam parameters Treatment protocols must be standardized before the true efficacy of laser therapy can be accurately assessed. Table 1 allows a comparison between several published treatment protocols. The aim of this study was to explore the usefulness of this laser by measuring its fungicidal effect in toenails diagnosed as cases of onychomycosis. We also determined the optimal conditions to treat onychomycosis by laser to achieve maximal clinical effect.

**Table 1:** Comparison of treatment parameters reported in different articles using 1064-nm laser

Frequency (Hertz)	Fluence (J/cm^2^)	Power	Pulse (ms)	Spot (mm)	Sweeps	Total number of sessions	Sessions/week	Ref.
1	35–40	NR	35	4	3	4	1	17
NR	223	NR	NR	2	NR	2–3	3	22
1	240–324	NR	30	3	3	4	1	23
5	14	NR	0.3	5	NR	3–4	4–8	24
20	250	5 W	0.1	NR	NR	NR	NR	25
5	14	NR	NR	2.5	NR	2	4	26
5	5	NR	0.3	6	NR	NR	NR	27
NR	50	NR	40	3	NR	2	4	28

Moreover, the effect of laser on fungal morphology was assessed by atomic force microscopy (AFM).

## Materials and methods

The study series consisted of 156 patients with clinically diagnosed onychomycosis. All patients were recruited at the Podiatry Hospital of the University of Barcelona between September 2012 and March 2013. The research was previously approved by the “Comissio de Bioética de la Universitat de Barcelona”.

- Inclusion criteria: Patients had to be older than 18 years, with one or more nails affected by any of the clinical presentations of onychomycosis. A signed informed consent document was required from all participants.
- Exclusion criteria: Patients treated with antifungal agents, either topical or systemic, during the previous 6 months, or under photosensitizing treatment, or with subungual hematomas, hyperpigmentation of the nail bed, nail bed disturbance, or discoloration irrespective of cause; pregnant women were excluded. Moreover, since level of pigmentation could imply significant differences in the energy adsorption, all subjects included fell into the groups of pigmentation I to IV of the widely used Fitzpatrick classification [21]. No individuals of groups V and VI were included in the study.
- Procedure: In patients eligible for the study, a control photograph of the affected nail(s) was taken and the following data were recorded: job, sports activities, complementary activities, personal and/or family history of onychomycosis, pets, whether the patient walked barefoot and where, and the number of affected nails. Onychomycotic nails were cleaned with alcohol and then trimmed, which improved sampling of the most highly involved area (where fungal activity was more apparent). Nails severely dystrophic or onychogryphotic (abnormal hypertrophied and curved) were subjected to comprehensive milling. The patient was then seen 1 month later, for culture confirmation of a mycotic infection and treatment thereof.

Laboratory diagnosis was performed by microscopic examination of nail samples treated with 40% potassium hydroxide. Whereas the observation of fungi was considered indicative of infection, a negative result did not rule out infection. Thus, all samples of suspected cases were cultured regardless of the result of the direct examination. Four affected nail fragments were inoculated on Sabouraud medium with chloramphenicol and four on medium with cycloheximide. The cultures were incubated at 28–30 °C for 4–6 weeks and examined once a week until the colonies were visualized. The fungi were stained with lactophenol blue and microscopically observed. If only filamentous, non-dermatophyte fungi were found, a new sample was collected from the respective patient.

The culture plates were then incubated for an additional week at room temperature to allow sporulation. In some cases, potato dextrose agar was used to stimulate spore formation. The macroscopic and microscopic characteristics of the macroconidia and microconidia were used to identify the fungi. To differentiate between *Trichophyton mentagrophytes* complex and *Trichophyton rubrum*, urease activity was determined.

Yeast identification was carried out using chromogenic culture medium (Brilliance *Candida* agar, Oxoid), the horse serum filamentation test, and, when necessary, the API-C (Biomerieux) auxonogram test.

On the second visit, we proceeded to treat the infected toenails with 1064-nm laser, using a Podylas S30 (INTERmedic; Cerdanyola (Barcelona), Spain) laser and the following parameters: frequency: 1 Hz, fluence 35–40 J/cm^2^, power 30 W, sweeping an affected area 3 mm in diameter. Variations in fluence were selected based on the thickness of the toenail (thicker toenails require greater fluence) as well as the heat sensitivity of the patient. To optimize treatment and cover the complete nail surface, three passes were performed, with each pass consisting of a longitudinal and a transversal sweep, including the eponychium and the nail channels. As a preventive measure, healthy nails of all patients were subjected to one pass with the laser at the same settings. To ensure that the nail temperature did not exceed 47 °C ± 4, an external thermal camera was used continuously. The three treatment sessions were separated by an interval of 15 days each. Appointments to follow up patients were scheduled 3, 6, 9, 12, 14, and 18 months after the first visit. Visits were ended when complete healing was achieved; the length of the follow-up period strongly depended upon the clinical presentation (see below). During those visits, the treated area was examined for eventual side effects, the degree of patient satisfaction was determined, and a photograph of the treated nail(s) was taken. When healing defined as the disappearance of symptoms was observed, a new culture was performed to confirm fungal negativity. During the course of treatment, the patients were instructed to follow several prophylactic measures to avoid recurrence: inspection of potentially contaminated footwear and disposal of the oldest footwear (possible source of self-contagion), application of antifungal powder in all footwear to be worn during treatment, and disinfection of pedicure utensils. Basic hygiene rules were prescribed: the use of an acidic (5.5) pH soap in daily hygiene of feet thorough drying with unshared towels, no sharing of footwear, and the avoidance of areas of potential contamination in order to prevent reinfection.

### In vitro experiments: light source and irradiance parameters

The same equipment was used to irradiate microconidia according to the parameters used in the daily practice; a 1064-nm wavelength laser set at 30 W and an energy of 35 J/cm^2^ was used to scan entire mica slide after placing microconidia. This procedure was repeated up to three times, following the same methodology as in nail’s treatment described above. The samples were afterwards immediately imaged by atomic force microscopy.

### AFM imaging

AFM measurements were performed in air with a XE-70 (Park Systems, South Korea) microscope at room temperature in non-contact mode using a silicon cantilever ACTA (Applied NanoStructures, CA, USA) with a nominal resonance frequency of 300 kHz and a nominal force constant of 37 N/m. *T. rubrum* spores were obtained by fungal cultivation on Sabouraud dextrose agar medium (Oxoid, UK) at 30 °C for 14 days. A volume of 8 mL of phosphate-buffered saline (NaCl 80 g, KCl 2 g, Na_2_HPO_4_ 14.2 g, KH_2_PO_4_ 2.7 g in 1 l) was then poured onto plates containing 0.01% Tween 80, and the surface was scraped with an L-shaped spreader in order to release the microconidia. Density of the spore suspensions was monitored by using a Neubauer chamber. Suspension was stored at 4 °C until use. Samples were imaged by spot inoculating 10 μl of the microconidial suspension onto mica slides and left to air-dry. Measurements began by scanning a random area of 30 × 30 μm, which was gradually decreased until bacterial surface could be observed in detail. Topography, amplitude, and phase images (5 × 5 μm) were recorded simultaneously.

## Results

Of the 156 processed samples, 119 (76%) gave positive microbiological culture results at baseline. The affected nails of the respective patients were treated in three sessions. Three patients withdrew from the study. Of the 119 positive nails, 68 were on the right foot and 51 on the left foot; 110 of the infected nails involved the first toe, three the second, four the third, and two the fourth. The extent of infection was as follows: 41 totally affected nails, 27 nails in which approximately two thirds was affected, 23 nails with one third affected, and 28 nails distally infected. The distribution of the clinical presentation was as follows: distal and lateral subungual onychomycosis (*n* = 74), totally dystrophic onychomycosis (*n* = 41), proximal subungual onychomycosis (*n* = 2), endonyx onychomycosis (*n* = 1), and superficial onychomycosis (*n* = 1).

Microbiological culture gave the following results: *T. rubrum* (*n* = 61 cases), *T. mentagrophytes* (*n* = 13), *Trichophyton tonsurans* (*n* = 13), *Scopulariopsis spp*. (*n* = 9), *Aspergillus spp.* (*n* = 7), *Rhodotoroula spp.* (*n* = 6), *Acremonium spp.* (*n* = 5), *Trichophyton interdigitale* (*n* = 4), *T. violaceum* (*n* = 2), *Microsporum spp*. (*n* = 2), *Candida parapsilosis* (*n* = 2), *Cryptococcus laurentii* (*n* = 2), *Candida famata* (*n* = 1), *Alternaria spp*. (*n* = 1), and *Cryptococcus spp.* (*n* = 1).

Patients did not refer explicitly to pain during treatment. In the few patients who mentioned a transient sensation of heat, treatment was interrupted for a few minutes and the area was cooled with gauze soaked in saline solution. There have been no reports of side effects as a result of treatment.

**Fig. 1:**
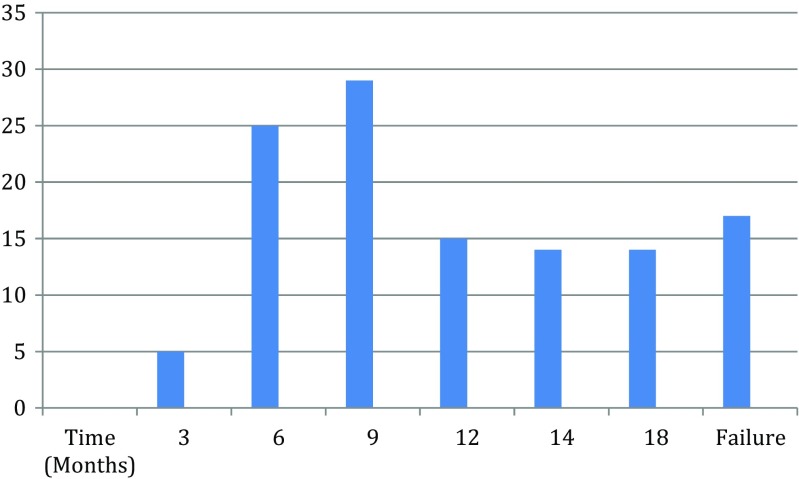
Distribution of percentages of cases cured at different periods; 15.3% were considered as non-cured

The clinical evolution of the patients included in this study was as follows: after 3 months, five patients were completely symptom-free and their microbiological cultures were negative. In 25 patients, negative cultures and the absence of symptoms was achieved after 6 months of treatment; in 29 patients, 9 months were required and the remaining patients were followed for more than 1 year as can be seen in Fig. 1. All cured patients reported a high degree of satisfaction and did not report secondary effects. Moreover, accomplishment of the treatment prescriptions (mostly hygiene) resulted easy for all patients included in the study. Results are summarized in Table 1. It should be emphasized that clinical success did not depend upon the etiological agent involved and that depicted signs, allowing picture definition, were related to the length of treatment period; thus, total dystrophic nails require the longest periods. In all cases defined as clinical healing, the last culture was negative.

**Fig. 2:**
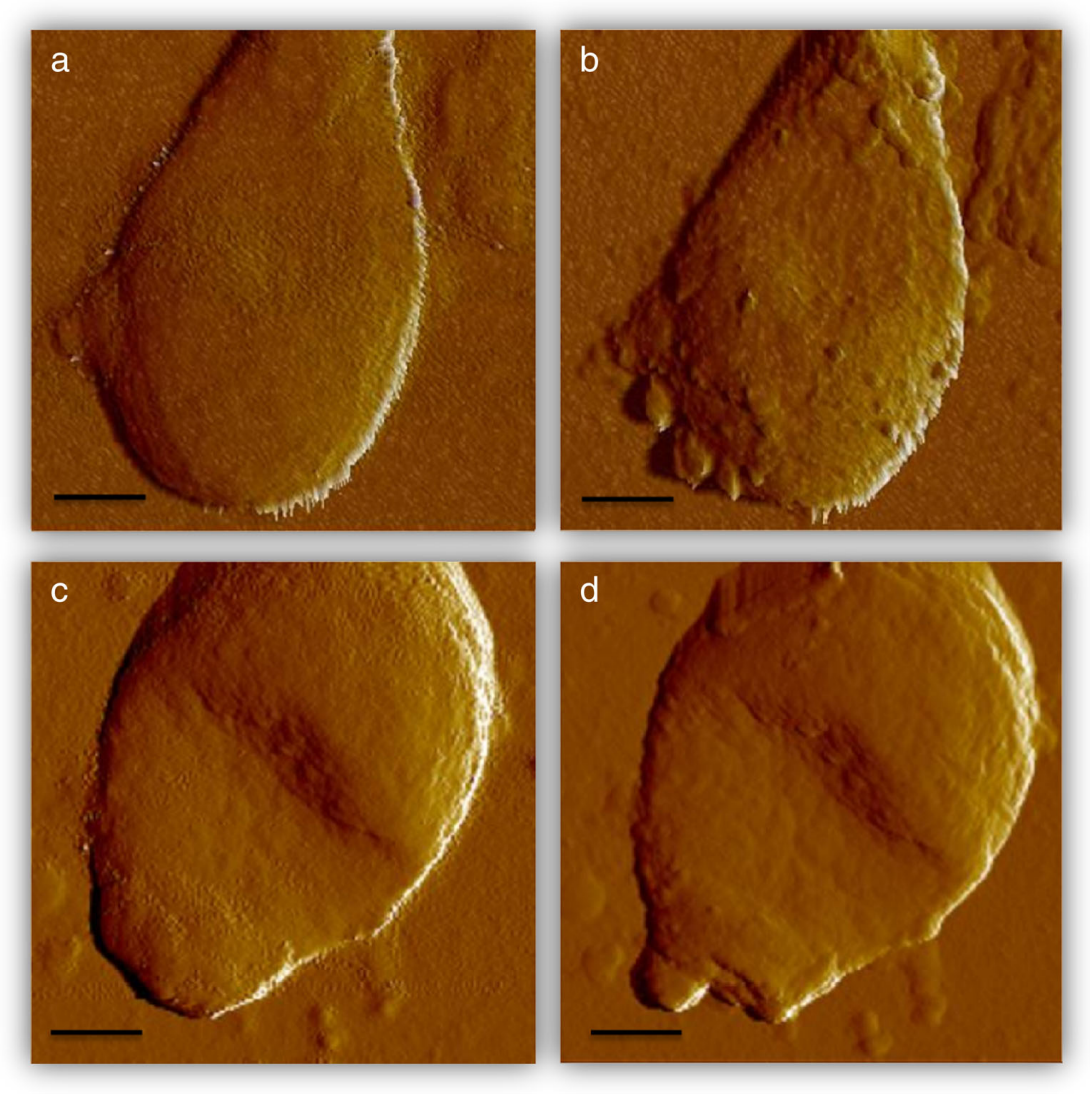
Error signal measurements performed in two different microconidia, previous to laser treatment (**a**, **c**) and after three sessions at with a 1064-nm wavelength laser at 40 W and 35 J/cm^2^ (**b**, **d**). *Scale bar* 1 μm

**Fig. 3:**
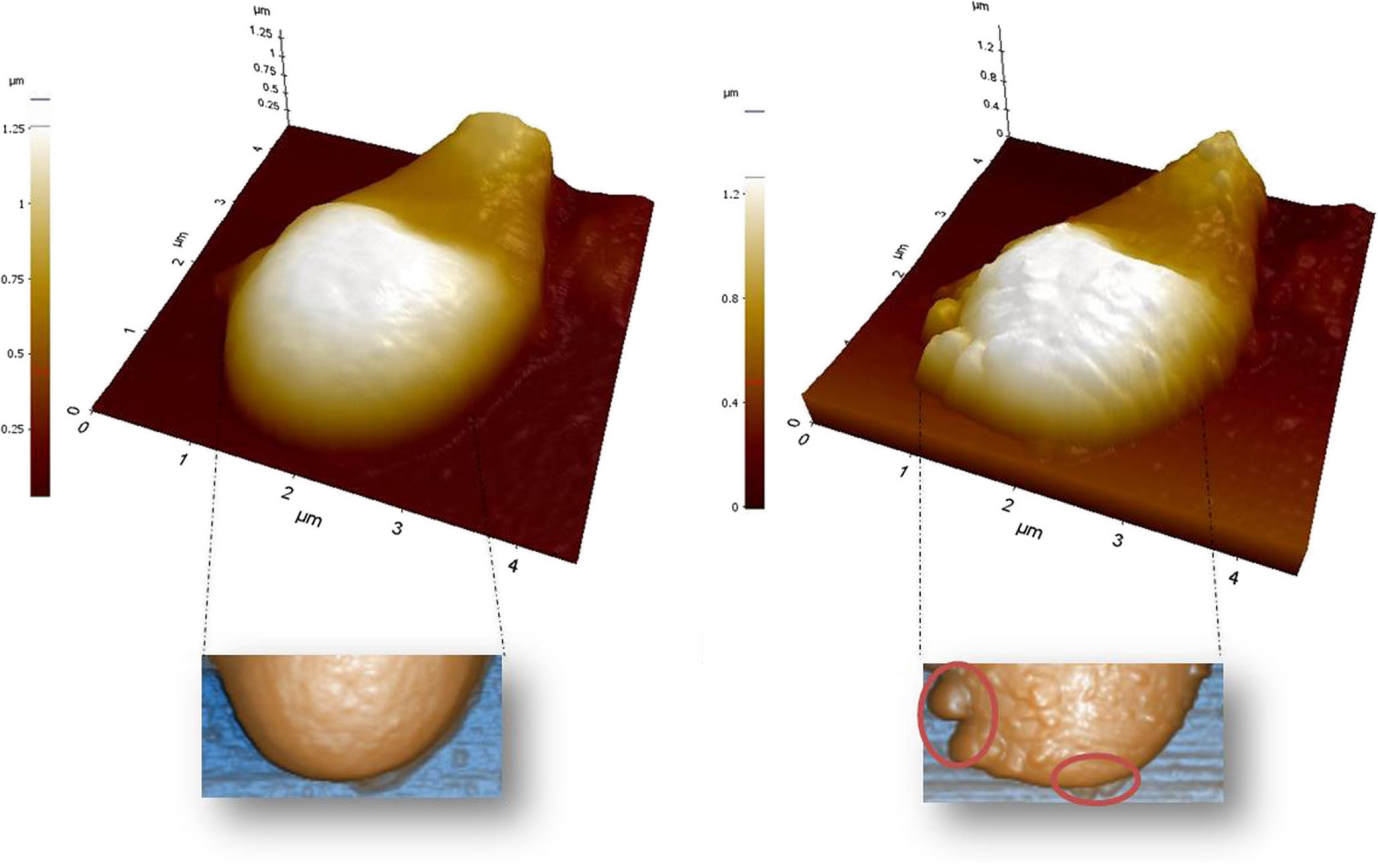
Topography images corresponding to Fig. 2a, b, under the same conditions as described previously

Error signal measurements performed in two different microconidia, previous to laser treatment (a and c) and after three sessions at with a 1064-m wavelength laser at 40 W and 35 J (b and d), are shown in Fig. 2, while topography images corresponding to Fig. 2a, b under the same conditions as described previously are shown in Fig. 3.

## Discussion

Onychomycosis of the toenails differs from that involving the fingernails, mostly because patients do not immediately notice that their toenails have undergone morphological or color changes but also because occlusive footwear and socks generate environmental conditions in which frequent minor injuries to the nail bed are likely. The presence of vascular diseases is also an important consideration, as these often promote dystrophic or thickened nails, in turn favoring the development of fungal infections. Individuals with diabetes, immunosuppressive pathologies, or undergoing medical treatment leading to immunological impairment are also highly susceptible to mycotic diseases, including onychomycosis.

The treatment of onychomycosis of the toenails is hindered not only by the concomitant thickening of the nail, which slows or even prevents the penetration of topical antifungals, but also by the poor vascularization of the toes, as commonly occurs in patients with the abovementioned pathologic conditions. Fungal infections of the toenails are mainly caused by dermatophytes such as *T. rubrum*, *T. mentagrophytes*, *T. tonsurans*, and *Microsporum*, but in many cases, non-dermatophyte filamentous fungi are isolated, such as *Scopulariopsis*, *Acremonium*, *Alternaria*, and yeasts of the *Candida* genus, such as *C. parapsilosis* and *C. famata* or of the *Rhodotoroula* genus. This variety of pathogens poses further obstacles to effective treatment and highlights the importance of a correct laboratory-based diagnosis of the causative agent. The significance of the isolation of non-dermatophyte filamentous fungi is unclear, although as supported by our study, their presence has been associated with abnormal nail development [22].

The appropriateness and usefulness of the treatment of onychomycosis, whether by topical and oral pharmacologic agents, photodynamic therapy, iontophoresis, or laser, have been discussed in literature reviews [23, 24]. Topical pharmacologic compounds are effective in distal onychomycosis, although the thickness of the nail plate makes their penetration difficult. Thus, in patients treated with topical antifungals, the affected nail plate should be milled to diminish its thickness and thereby to facilitate drug activity. Systemic treatment of onychomycosis is limited by the toxicity, side effects, and adsorption limitations of oral agents and by a lack of agreement as to the most effective treatment strategy. However, among clinicians, it is widely accepted that oral antifungal treatment can be administered when two thirds of the nail is involved. Nonetheless, certain patients with onychomycosis cannot be treated with oral agents because of other, underlying pathologies. Combined topical and oral treatments are recommended when there is a generalized involvement of the nail [25], discontinuing the oral agent as soon as possible, whereas, depending on the characteristics of the pathology, topical treatment may be prolonged for 4–6 months.

Using the protocol described herein, we were able to treat our patients without the need for any form of analgesia. It has been shown that lower potencies and lesser number of sessions could lead to negative results [26]; in this work, we obtained satisfactory results with our procedure. A combination of laser and topical treatment [27] has been suggested for patients in whom systemic antifungal agents are contraindicated and for patients with diabetic neuropathies. However, in both groups, extreme caution is required, since the lack of pain sensation, typical in these patients, can lead to serious injury. It has been pointed out that despite early data are promising, many of studies dealing with onychomycoses treatment by laser are small or poorly designed [28]; thus, this work attempts to contribute to a major extent of this research. Moreover, injuries in fungal structure induced by laser treatment can be easily observed by AFM visualization. Laser treatment originates discontinuities in cell envelopes allowing the cytoplasmic content to leak.
